# Complete Genome Sequence of a Colistin-Resistant *Escherichia coli* Strain Harboring *mcr-1* on an IncHI2 Plasmid in the United States

**DOI:** 10.1128/genomeA.01095-17

**Published:** 2017-10-19

**Authors:** Victoria L. Gilrane, Stephen Lobo, Weihua Huang, Jian Zhuge, Changhong Yin, Donald Chen, Kimberly J. Alvarez, Alexandra Budhai, Ilana Nadelman, Nevenka Dimitrova, John T. Fallon, Guiqing Wang

**Affiliations:** a Department of Pathology, New York Medical College, Valhalla, New York, USA; b Department of Pathology and Clinical Laboratories, Westchester Medical Center, Valhalla, New York, USA; c Department of Medicine, New York Medical College, Valhalla, New York, USA; d Department of Infection Prevention and Control, Westchester Medical Center, Valhalla, New York, USA; e Metropolitan Area Regional Office, New York State Department of Health, New Rochelle, New York, USA; f Philips Research North America, Cambridge, Massachusetts, USA

## Abstract

We report here the incidental detection and complete genome sequence of a urinary *Escherichia coli* strain harboring *mcr-1* and resistant to colistin in a New York patient returning from Portugal in 2016. This strain, with sequence type 1485 (ST1485), was a non-extended-spectrum beta-lactamase (ESBL) and non-carbapenemase producer and carried the *mcr-1* gene on an IncHI2 plasmid.

## GENOME ANNOUNCEMENT

The emergence of plasmid-mediated colistin resistance, associated with the *mcr-1* gene, was first reported in China in November 2015 (1). This gene has now been detected in more than 30 countries worldwide (2). As of 1 June 2017, 14 human cases have been reported from 9 U.S. states (https://www.cdc.gov/drugresistance/tracking-mcr1.html). Here, we report the complete genome sequence of an *mcr-1*-carrying *Escherichia coli* strain, which was incidentally detected from a New York state patient returning from Portugal in 2016.

The patient, a 51-year-old woman with history of ovarian cancer, was admitted in November 2016 for right lower extremity cellulitis and an upper respiratory tract infection. Three sets of blood cultures were negative for bacteria and fungi. A urine culture collected on the day of admission grew a non-extended-spectrum beta-lactamase (ESBL)- and non-carbapenemase-producing *E. coli* (strain M160133), which was incidentally included in a prospective surveillance study for *mcr-1* and was confirmed to carry *mcr-1* by an in-house real-time PCR assay. The patient had no history of receiving any polymyxins. She had traveled to the city of Coimbra, Portugal, in the summer of 2016 and stayed on a farm with chickens and pigs for 7 weeks.

*E. coli* strain M160133 was resistant to colistin, with an MIC of 4 µg/ml by Etest (bioMérieux, Durham, NC). Unlike other *mcr-1*-carrying *E. coli* strains reported in the United States, M160133 was resistant to ampicillin, ciprofloxacin, levofloxacin, tetracycline, and trimethoprim-sulfamethoxazole but was susceptible to the third- and fourth-generation cephalosporins, carbapenems, and tigecycline (3–8). The genome sequence was constructed on the basis of next-generation sequencing data from the MiSeq (Illumina) and RSII single-molecule real-time (SMRT) (Pacific Biosciences) systems, as described previously (9).

The complete genome of *E. coli* strain M160133 with sequence type 1485 (ST1485) consists of one 4.96-Mb chromosome and three plasmids with sizes of 233,149 bp (pM160133-p1), 173,624 bp (pM160133-p2), and 113,428 bp (pM160133-p3). Plasmid pM160133-p1 carries the *mcr-1* gene and belongs to incompatibility group IncHI2 (10). In addition to *mcr-1*, resistance genes *aadA2, dfrA12, floR*, *strA*, and *tet*(M) were also identified on this plasmid. Plasmid pM160133-p2 carried resistant genes *bla*_TEM-1B_,* dfrA14, sul2*, *strB*, and *tet*(A). No resistance genes were identified on plasmid pM160133-p3. Sequence BLAST analysis demonstrated that the IncHI2 plasmid pM160133-p1 shared >90% nucleotide identity to plasmids pHNSHP45-2 (*E. coli* isolate SHP45 from China, GenBank accession no. KU341381) (1), p14008_M1 (*E. coli* isolate NRZ14408 from Germany, accession no. LT599829), and pS38 (*E. coli* isolate S38, accession no. KX129782) (11).

In summary, we report the detection of a urinary *E. coli* strain harboring *mcr-1* and resistant to colistin in a New York patient returning from Portugal in 2016. This is the first report of a non-ESBL-producing non-carbapenemase-producing *E. coli* strain sheltering *mcr-1* on an IncHI2 plasmid in the United States, which raises a new challenge to current practice by testing isolates for *mcr-1*-mediated colistin resistance mainly in ESBL-producing *Enterobacteriaceae*.

### Accession number(s).

The complete genome sequence of strain M160133 has been deposited to GenBank under accession no. CP022164 for the chromosome, CP022165 for the *mcr-1*-carrying plasmid pM160133_p1, CP022166 for the pM160133_p2 plasmid, and CP022167 for the pM160133_p3 plasmid. These sequences are part of BioProject no. SAMN07273977.

## References

[1] Liu YY, Wang Y, Walsh TR, Yi LX, Zhang R, Spencer J, Doi Y, Tian G, Dong B, Huang X, Yu LF, Gu D, Ren H, Chen X, Lv L, He D, Zhou H, Liang Z, Liu JH, Shen J 2016 Emergence of plasmid-mediated colistin resistance mechanism MCR-1 in animals and human beings in China: a microbiological and molecular biological study. Lancet Infect Dis 16:161–168. 26603172 10.1016/S1473-3099(15)00424-7

[2] Giamarellou H 2016 Epidemiology of infections caused by polymyxin-resistant pathogens. Int J Antimicrob Agents 48:614–621. doi:10.1016/j.ijantimicag.2016.09.025. 27865627

[3] McGann P, Snesrud E, Maybank R, Corey B, Ong AC, Clifford R, Hinkle M, Whitman T, Lesho E, Schaecher KE 2016 *Escherichia coli* harboring *mcr-1* and *bla*_CTX-M_ on a novel IncF plasmid: first report of *mcr-1* in the United States. Antimicrob Agents Chemother 60:4420–4421. doi:10.1128/AAC.01103-16. 27230792 PMC4914657

[4] Kline KE, Shover J, Kallen AJ, Lonsway DR, Watkins S, Miller JR 2016 Investigation of first identified *mcr-1* gene in an isolate from a U.S. patient—Pennsylvania, 2016. MMWR Morb Mortal Wkly Rep 65:977–978. doi:10.15585/mmwr.mm6536e2. 27631164

[5] Mediavilla JR, Patrawalla A, Chen L, Chavda KD, Mathema B, Vinnard C, Dever LL, Kreiswirth BN 2016 Colistin- and carbapenem-resistant *Escherichia coli* harboring *mcr-1* and *bla*_NDM-5_, causing a complicated urinary tract infection in a patient from the United States. mBio 7(4):e01191-16. doi:10.1128/mBio.01191-16. 27578755 PMC4999550

[6] Macesic N, Green D, Wang Z, Sullivan SB, Shim K, Park S, Whittier S, Furuya EY, Gomez-Simmonds A, Uhlemann AC 2017 Detection of *mcr-1*-carrying *Escherichia coli* causing bloodstream infection in a New York City hospital: avian origins, human concerns? Open Forum Infect Dis 4. doi:10.1093/ofid/ofx115. PMC5508776 28721352

[7] Vasquez AM, Montero N, Laughlin M, Dancy E, Melmed R, Sosa L, Watkins LF, Folster JP, Strockbine N, Moulton-Meissner H, Ansari U, Cartter ML, Walters MS 2016 Investigation of *Escherichia coli* harboring the *mcr-1* resistance gene—Connecticut, 2016. MMWR Morb Mortal Wkly Rep 65:979–980. doi:10.15585/mmwr.mm6536e3. 27631346

[8] Zhu W, Lawsin A, Lindsey RL, Perry KA, Batra D, Rowe LA, Yoo BB, Lonsway D, Limbago B, Rasheed JK, Laufer Halpin A 2017 Whole genome sequencing and plasmid analysis of three *mcr-1* bearing *Escherichia coli* isolates from US patients, abstr 136 Microbe 2017, American Society for Microbiology, New Orleans, LA, 1–5 June 2017.

[9] Huang W, Wang G, Sebra R, Zhuge J, Yin C, Aguero-Rosenfeld ME, Schuetz AN, Dimitrova N, Fallon JT 2017 Emergence and evolution of multidrug-resistant *Klebsiella pneumoniae* with both *bla*_KPC_ and *bla*_CTX-M_ integrated in chromosome. Antimicrob Agents Chemother 61:e00076-17. doi:10.1128/AAC.00076-17. 28438939 PMC5487645

[10] Carattoli A, Zankari E, García-Fernández A, Voldby Larsen M, Lund O, Villa L, Møller Aarestrup F, Hasman H 2014 *In silico* detection and typing of plasmids using PlasmidFinder and plasmid multilocus sequence typing. Antimicrob Agents Chemother 58:3895–3903. doi:10.1128/AAC.02412-14. 24777092 PMC4068535

[11] Zurfluh K, Klumpp J, Nüesch-Inderbinen M, Stephan R 2016 Full-length nucleotide sequences of *mcr-1*-harboring plasmids isolated from extended-spectrum-beta-lactamase-producing *Escherichia coli* isolates of different origins. Antimicrob Agents Chemother 60:5589–5591. doi:10.1128/AAC.00935-16. 27324774 PMC4997865

